# Impact of 3D Printing on Short-Term Outcomes of Biventricular Conversion From Single Ventricular Palliation for the Complex Congenital Heart Defects

**DOI:** 10.3389/fcvm.2021.801444

**Published:** 2021-12-21

**Authors:** Bozhong Shi, Yanjun Pan, Weiru Luo, Kai Luo, Qi Sun, Jinlong Liu, Zhongqun Zhu, Hao Wang, Xiaomin He, Jinghao Zheng

**Affiliations:** ^1^Department of Cardiothoracic Surgery, Shanghai Children's Medical Center Affiliated to Shanghai Jiao Tong University School of Medicine, Shanghai, China; ^2^Shanghai Children's Medical Center, Institute of Pediatric Translational Medicine, Shanghai Jiao Tong University School of Medicine, Shanghai, China

**Keywords:** complex congenital heart defects, biventricular conversion, 3D printing, surgical outcomes, pre-operative evaluation

## Abstract

**Background:** Although Fontan palliation seems to be inevitable for many patients with complex congenital heart defects (CHDs), candidates with appropriate conditions could be selected for biventricular conversion. We aimed to summarize our single-center experience in patient selection, surgical strategies, and early outcomes in biventricular conversion for the complex CHD.

**Methods:** From April 2017 to June 2021, we reviewed 23 cases with complex CHD who underwent biventricular conversion. Patients were divided into two groups according to the development of the ventricles: balanced ventricular group (15 cases) and imbalanced ventricular group (8 cases). Early and short-term outcomes during the 30.2 months (range, 4.2–49.8 months) follow-up period were compared.

**Results:** The overall mortality rate was 4.3% with one death case. In the balanced ventricular group, 6 cases received 3D printing for pre-operational evaluation. One case died because of heart failure in the early postoperative period. One case received reoperation due to the obstruction of the superior vena cava. In the imbalanced ventricular group, the mean left ventricular end-diastolic volume was (33.6 ± 2.1) ml/m^2^, the mean left ventricular end-diastolic pressure was 9.1 ± 1.9 mmHg, and 4 cases received 3D printing. No death occurred while one case implanted a pacemaker due to a third-degree atrioventricular block. The pre-operational evaluation and surgery simulation with a 3D printing model helped to reduce bypass time in the balanced group (*p* < 0.05), and reduced both bypass and aorta clamp time in the imbalanced group (*p* < 0.05). All patients presented great cardiac function in the follow-up period.

**Conclusion:** Comprehensive evaluation, especially 3D printing technique, was conducive to finding the appropriate cases for biventricular conversion and significantly reduced surgery time. Biventricular conversion in selected patients led to promising clinical outcomes, albeit unverified long-term results.

## Introduction

The intricate variation of intracardiac anomalies and imbalanced ventricular development largely restrain the surgical choices for complex congenital heart defects (CHDs). To a great extent, single ventricular palliation is traditionally a preferred option for neonates with complex CHDs (1–3). Owing to its feasibility and lower early complications, it is undeniable that the short- to medium-term outcomes of Fontan palliation is somehow satisfying (4). However, in the long-term perspective, Fontan physiology not only exerts its influence on the circulatory system *per se* but also hampers extracardiac development in a systemic view. A large number of patients with single ventricular circulation experience poor life quality due to various progressive and burdensome complications accumulated with time, which remains its inherent problem (5–8).

Fortunately, with great advances in both surgical concepts and techniques over the years, a subset of patients with complex CHDs could be selected to undergo biventricular conversion in a few experienced centers. To avoid the drawbacks of Fontan circulation and in pursuit of better long-term life quality, previous cavopulmonary connections are taken down and biventricular circulation is surgically restored. Although no criteria have yet been established regarding patient selection and the long-term outcomes are still unwarranted, recent studies have reported the favorable short-term results of biventricular conversion, giving great promise to this new enthusiasm (9–12). Moreover, the new emerging three-dimensional (3D) printing technique provides intuitionistic information on cardiac structures, allowing us to accurately select the most suitable patients and set surgical plans (13, 14). Here, we described and summarized our single-center surgical strategies of biventricular conversion from Fontan palliation for patients with complex CHDs, and we also hypothesized and depicted that the 3D printing technique was helpful in pre-operational evaluation.

## Methods

### Study Population

**Figure 1:**
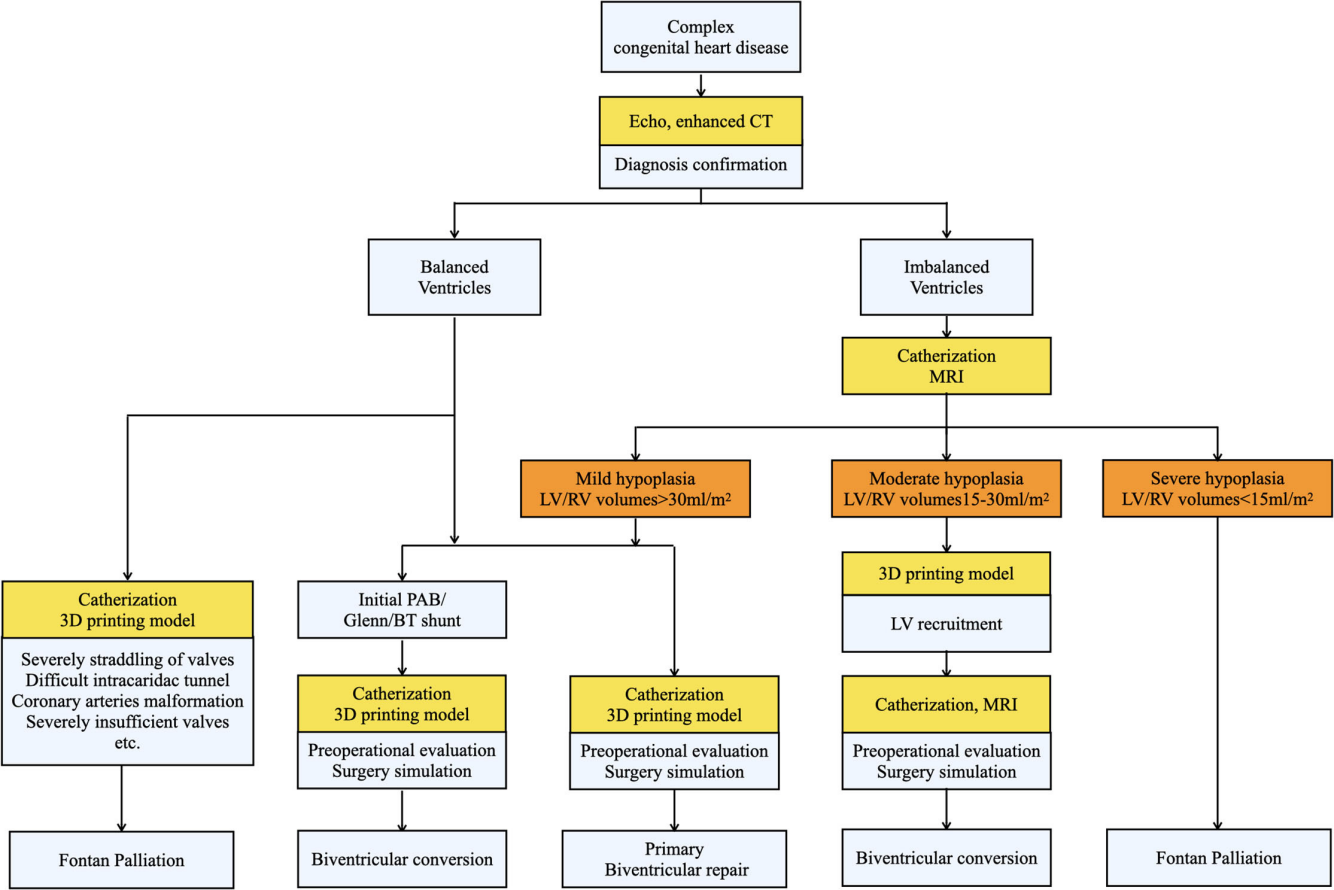
The surgical decision-making process of biventricular conversion in our center. Patients were classified into balanced and imbalanced groups after being diagnosed by echocardiography and enhanced CT. Then various evaluations (shown in yellow) were conducted to determine the feasibility of biventricular correction and different strategies were accordingly applied.

From April 2017 to June 2021, we retrospectively reviewed 23 patients in Shanghai Children Medical Center, who initially planned to receive Fontan operation but converted to biventricular correction after evaluation. The ethical approval was obtained by Shanghai Children Medical Center Ethics Committee. To determine the feasibility of biventricular conversion, all patients were meticulously examined before surgical decisions. Echocardiography was used to determine cardiac anomalies and confirm the diagnosis. Enhanced cardiac CT and/or cardiac MRI were employed to assess ventricular mass and ventricular development. Further cardiac catheterization and angiography were conducted to assess hemodynamic parameters when necessary. The patient selection, surgical choice, and definition of ventricular hypoplasia in our study were generated mainly from the experience of the Boston Children's Hospital (9–11). A detailed surgical decision-making process was illustrated in Figure 1. Based on the development of two ventricles and anatomic characteristics, our 23 patients were divided into 2 groups: the balanced ventricular group (15 cases) and the imbalanced ventricular group as defined by left ventricular end-diastolic volume (LVEDV) <40 ml/m^2^ measured in MRI (8 cases). The hypoplasia of the ventricle was further classified by MRI into mild hypoplasia (LVEDV >30 ml/m^2^), moderate hypoplasia (LVEDV 15–30 ml/m^2^), and severe hypoplasia (LVEDV <15 ml/m^2^) (9), which is shown in Figure 1 and stood as a major standard for surgical decision. Moreover, 6 patients in the balanced group and 4 patients in the imbalanced group also received cardiac 3D printing for a more visualized and detailed evaluation. The baseline characteristics are shown in Table 1.

**Table 1:** Baseline characteristics stratified by different groups.

	**Balanced ventricular group (*n* = 15)**	**Imbalanced ventricular group (*n* = 8)**	***P*-value**
Gender (male/female)	12/3	4/4	0.136
Age at primary surgery (month)	8.6 ± 5.2	10.2 ± 5.6	0.604
Weight at primary surgery (kg)	6.3 ± 2.6	7.9 ± 3.9	0.358
Waiting time for BC (month)	56.4 ± 38.9	20.4 ± 15.8	0.074
Age at BC (month)	65.0 ± 37.9	30.6 ± 16.8	0.081
Weight at BC (kg)	17.0 ± 4.8	13.4 ± 4.0	0.182
LVEF(%)	57.1 ± 5.9	60.2 ± 4.6	0.332

*BC, biventricular conversion; LVEF, left ventricle ejection fraction*.

### 3D Printing Heart Model

Enhanced CT images of selected patients were first acquired and exported as Digital Imaging and Communications in Medicine format for the latter process. The highly integrated medical image processing software, Materialise-Mimics Innovation Suite 19.0 (Materialise NV., Leuven, Belgium) was utilized to segment the targeted cardiac blood flow areas, and Materialise-3-Matic 12.0 (Materialise NV., Leuven, Belgium) was used for smoothing the numerical vascular models. Before sending to the 3D printer, these reconstructed models were saved in stereolithography (STL) interface format. All these reconstruction processes were performed by experienced engineers under the guidance of cardiac surgeons. All these 3D models were fabricated in the 3D printer (BQ Witbox, Spain) for clinical surgery applications.

### Surgical Techniques for Biventricular Conversion

For patients with balanced, two well-developed ventricles, the operation was performed first by taking down the previous Glenn, and/or Blalock–Taussig shunt, and/or pulmonary artery banding (PAB). The following biventricular correction was performed according to different intracardiac abnormalities. The intracardiac tunnel was established to connect ventricular septal defect (VSD) and pulmonary artery or aorta according to the distance and relationship between VSD and these two major arteries. The right ventricular outflow tract was reconstructed by a transannular patch or conduit. Then Rastelli procedure, arterial switch operation (ASO), or double switch (DS) operation were accomplished. If Glenn was previously performed, after taking down the Glenn, the continuity of systemic venous pathway was reconstructed by anastomosing the posterior wall of the superior vena cava with the right atrial appendage and by a patch augmentation of the anterior wall of the superior vena cava to the right atrial junction.

For patients with imbalanced ventricles, an adequate evaluation of ventricular hypoplasia was carried out before surgery. To evaluate the feasibility of biventricular conversion in the imbalanced ventricular group, echocardiography was first used to confirm the diagnosis, then combined with MRI to ensure an adequate valve size and function (*Z*-scores > −2), the systolic and diastolic functions, and chamber size (LVEDV >30 ml/m^2^ for primary conversion and 15–30 ml/m^2^ for LV training) (9). Finally, catheterization was performed to ensure a relatively low left ventricular end-diastolic pressure (LVEDP) (<13 mmHg) of the ventricles to evaluate loading conditions after conversion. 3D printing was performed as well for visualized evaluation and pre-operative simulation. In patients with an EDV of 15–30 ml/m^2^ of the hypoplastic ventricle, LV recruitment by a 4–5 mm ASD fenestration was performed as an attempt to promote the development of the hypoplastic LV. The main surgical procedures of biventricular conversion included single ventricular septation, anatomic repair of corresponding intracardiac malformations, Glenn and/or banding take-down, reconnection of superior vena cava and right atrium, and pulmonary artery augmentation.

### Follow-Up

All patients were followed up at 1, 3, 6, 9, and 12 months after discharge and once a year thereafter. Patients were analyzed for general condition and echocardiography at each time. The early mortality, postoperative complications, cardiac function, and reoperation/reintervention of the two groups were compared and analyzed.

### Statistical Analysis

IBM SPSS Statistics version 22.0 (IBM-SPSS Inc, Armonk, NY, United States) was used for statistical analysis. The continuous variables were expressed by mean ± SD, and the comparison of which between two groups was conducted by *t*-test. Categorical variables were reported in the form of the frequency with percentage (%) and the comparison was made by chi-square test. *P*-values <0.05 were considered statistically significant.

## Results

### Pre-operational 3D Printing Evaluation

When the decision of whether to perform biventricular conversion was hard to make due to limited information provided by traditional flat images (echocardiography, CT, and MRI), we employed a 3D printing heart model as an adjunction. Six patients in the balanced ventricular group and 4 patients in the imbalanced ventricular group received 3D printing before the operation, which was used to determine the key factors of biventricular conversion. Specifically, the key factors included the ventricular volume, VSD location and its distance with large vessels, infundibular range, and the construction of intraventricular channel. In both groups, the patients who received 3D printing evaluation and pre-operational simulation underwent a smooth and successful operation, presenting excellent early outcomes with no mortality and morbidity. Moreover, as shown in Table 2 in comparison to the patients with and without 3D printing, we found that 3D printing significantly reduced aortic clamp time in the balanced ventricular group (*p* < 0.05). In the imbalanced ventricular group, pre-operational simulation by 3D printing helped to reduce both bypass duration and clamp time (*p* < 0.05), indicating its prominent benefit.

**Table 2:** Surgery time of patients with and without 3D printing in two groups.

**Groups**	**Balanced ventricular group**			**Imbalanced ventricular group**		
**3D printing**	**+ (*n =* 6)**	**– (*n =* 9)**	***P*-value**	**+ (*n =* 4)**	**– (*n =* 4)**	***P*-value**
By-pass time (min)	171.5 ± 62.6	233.4 ± 65.6	0.091	140.3 ± 11.1	214.5 ± 57.0	**0.043**
Aortic cross-clamp time (min)	94.8 ± 43.1	145.9 ± 40.8	**0.037**	80.5 ± 20.0	138.3 ± 36.7	**0.033**

*Statistical significance was shown with bold values*.

**Figure 2:**
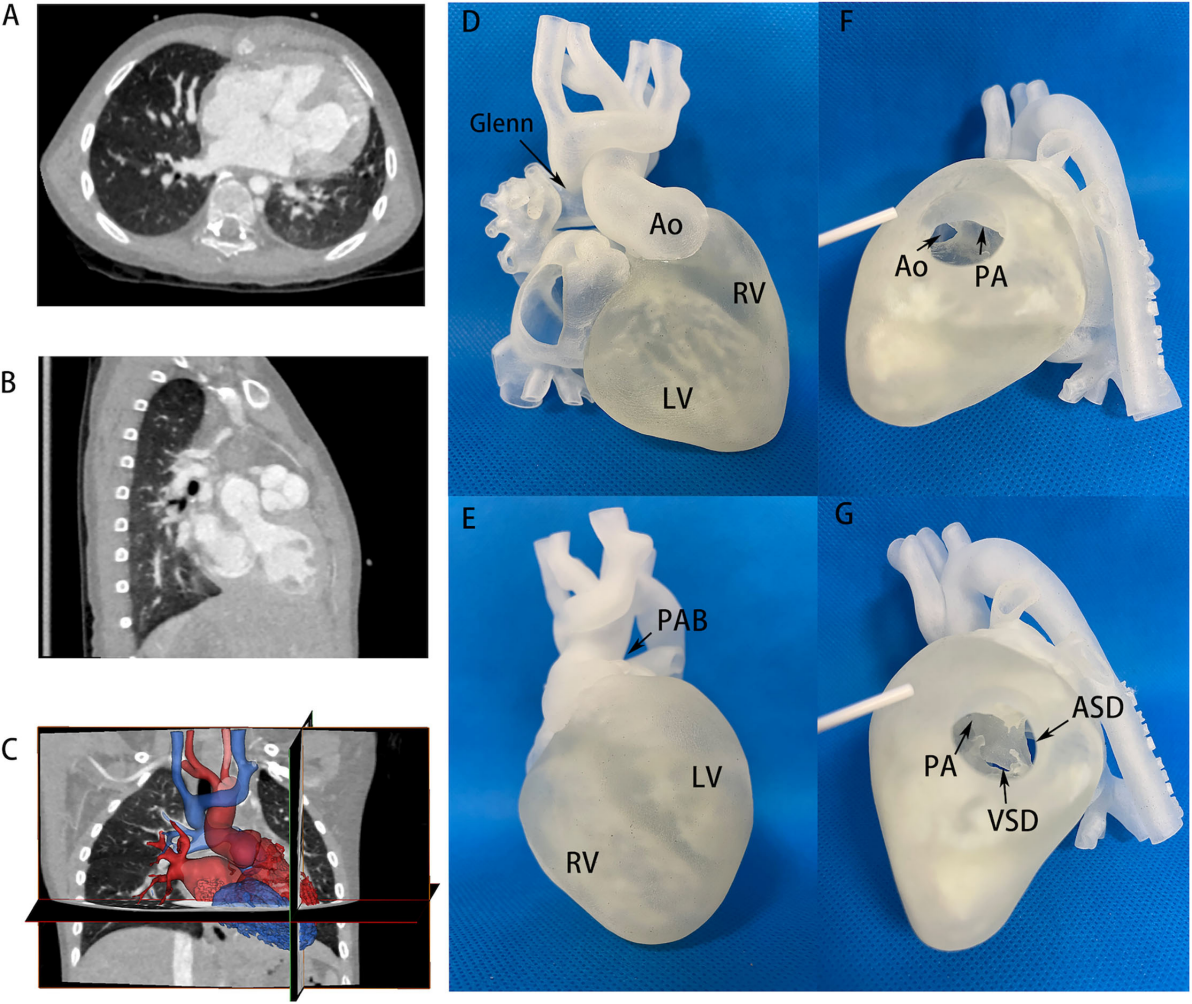
CT images and 3D printing model of case No. 3 in the balanced ventricular group. **(A,B)** CT images providing flat information of two well-developed ventricles, VSD size and location, atrioventricular discordance evidence. **(C)** Segmentation and reconstruction of the 3D model based on CT images, where red and blue color distinguished the two ventricles and circulation. **(D,E)** 3D printing models in anterior and inferior view, showing two well-balanced ventricles. The aorta and PA were in an anterior-posterior relationship. Glenn and PAB site in primary operation could also be seen. **(F,G)** 3D printing models on the right ventricular incision. Aorta and PA derived from right ventricles were observed. Ao, aorta; LV, left ventricle; RV, right ventricle; PA, pulmonary artery; PAB, pulmonary artery banding; VSD, ventricular septum defect; ASD, atrial septum defect.

**Figure 3:**
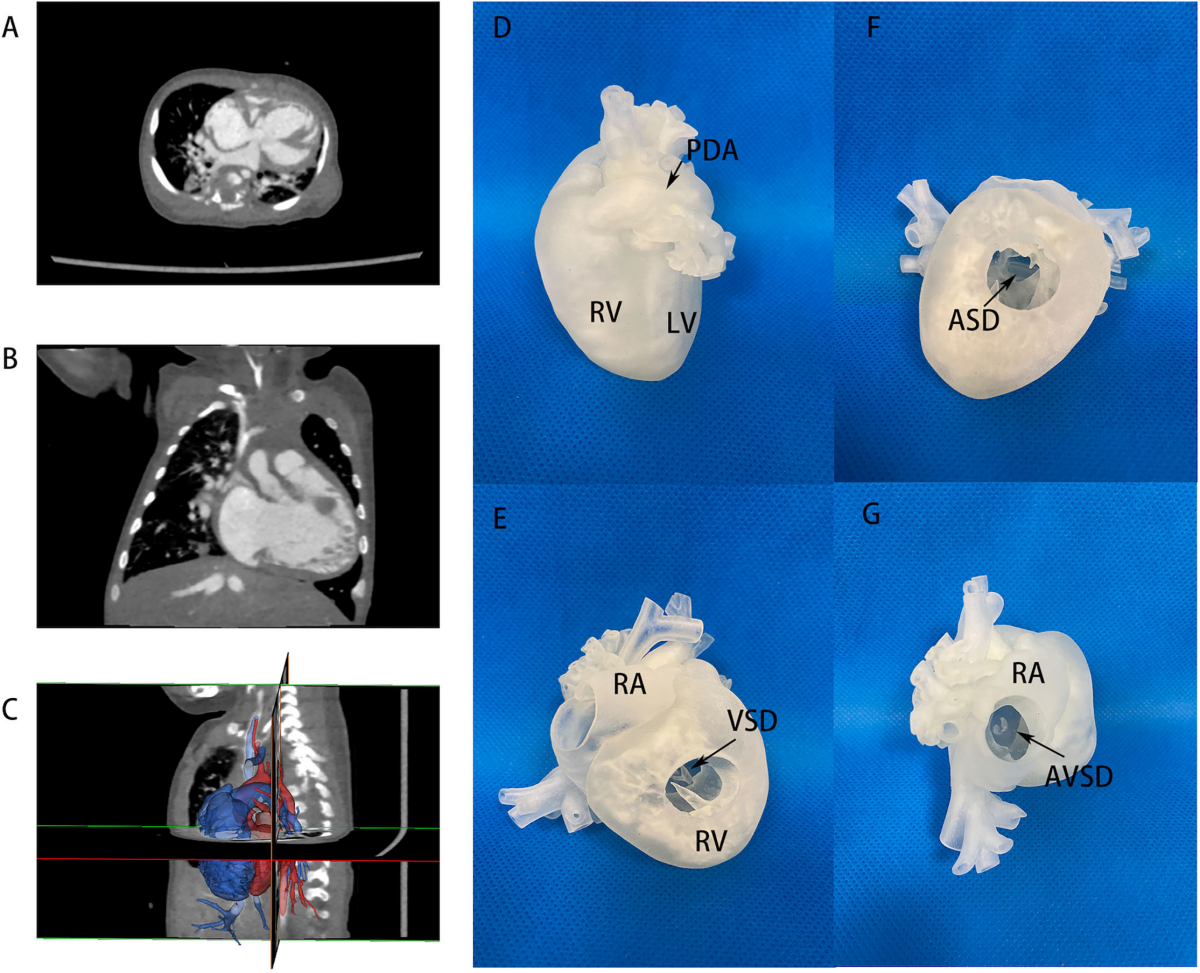
CT images and 3D printing model of case No. 17 in the imbalanced ventricular group. **(A,B)** CT images of two imbalanced ventricles and AVSD morphology, LV size was very small. **(C)** Segmentation and reconstruction of the 3D model, the lateral view showed a hidden, small LV posteriorly. **(D)** 3D printing models in lateral view, showing imbalanced ventricles. **(E)** 3D printing models in anterior view, VSD channel could be seen in RV incision. **(F)** ASD location *via* RV incision. **(G)** 3D printing models with RA incision. A large common atrioventricular channel was further evaluated. LV, left ventricle; RA, right atrium; RV, right ventricle; Ao, aorta; AVSD, atrioventricular septum defect; PDA, patent ductus arteriosus.

In addition, the 3D printing model played an important role in the change of surgical strategies. It provided visualized assistance to select proper patients for biventricular conversion who initially planned to receive univentricular palliation after traditional methods of evaluation. Figures 2, 3 demonstrate two representative examples in the balanced and the imbalanced groups, respectively. Case No.3 was a patient in the balanced ventricular group diagnosed with double outlet right ventricle (DORV) subpulmonary VSD, atrioventricular discordance, PDA. He received Fontan palliation as a first-stage operation. 3D printing model (Figure 2 and Supplementary Video 1) clearly showed a close distance and relationship of subpulmonary VSD and pulmonary artery, implying the feasibility of reconnection of the VSD to LV by patch repair. Thus, we decided to perform the biventricular conversion for the Fontan circulation to take place. A double switch (Mustard + ASO) operation and VSD closure were performed according to the pre-operational simulation on such a 3D model. The subaortic conus was removed. The previous PAB was taken down. The aorta and Pulmonary artery (PA) were translocated by Lecompt procedure. The VSD was repaired by pulmonary venous (PV) transvalvular dacron patch. Owing to pre-operational simulation on such a 3D model, biventricular conversion was achieved and the surgery went smoothly and successfully.

In the imbalanced ventricular group, a patient (case No. 17) diagnosed as AVSD with imbalanced ventricles was initially considered only suitable to Fontan palliation in another center due to an extremely small LV. The patient received 3D printing in our hospital to visualize the anatomic structure in detail and the actual volume of LV (Figure 3 and Supplementary Video 2). Cardiac catheterization showed an LVEDV of 17 ml/m^2^, also demonstrating the feasibility of a staged biventricular conversion. A first-staged PAB was performed to control pulmonary hypertension. A second-stage LV training including ASD repair with 5 mm atrial fenestration was done in the second-stage operation. After 5 months, cardiac catheterization and MRI showed that the LVEDP was 6 mmHg and LVEDV was 33 ml/m^2^. Based on such information, we believed that the left ventricle could withstand biventricular circulation at this point and performed successfully a biventricular conversion with AVSD correction in this patient.

### Early Postoperative Outcomes

A detailed illustration of all 23 cases, as classified into two groups, is shown in Tables 3, 4, including diagnosis, primary palliation, and final biventricular correction. There was one death case observed in a total of 23 patients, the overall mortality rate was 4.3%. Of the patients with balanced ventricles, the mean time interval between the first operation and biventricular conversion was 56.4 ± 38.9 months. One case died in early postoperative periods, who underwent IAA correction with pulmonary banding as primary stage operation, following another banding operation 2 years later, then received double switch operation (Senning + ASO) at last. Unfortunately, 2 weeks after the last operation, the patient presented a low cardiac output died from heart failure despite the application of ECMO. Concerning early complications in other cases, 2 patients developed an arrhythmia, 3 patients had pleural effusion, and 2 patients had a pulmonary infection.

**Table 3:** Operative data of each case receiving biventricular conversion in the balanced ventricular group.

**Case Number**	**Diagnosis**	**Primary operation**	**Biventricular conversion**
1	DTGA/VSD/PS/SA/PDA/single branch coronary artery	Right-side Glenn + PDA closure + MPA ligation	Rastelli + VSD closure + unclosed ASD + Glenn take-down
2	DORV remote VSD	Banding + ASD enlargement	Intracardiac tunnel + RVOT reconstruction + ASD closure + Banding take-down
3	DORV subpulmonary VSD/Atrioventricular discordance/PDA	Banding + Glenn + ASD enlargement + PDA ligation	Double Switch (Mustard + ASO) + VSD closure + Glenn take-down
4	DORV remote VSD/PS	Right-side Glenn	Intracardiac tunnel + RVOT reconstruction + ASD closure + Glenn take-down
5	PA/VSD	Right-side Glenn	PA/VSD repair + PFO repair + Glenn take-down
6	DORV remote VSD/ASD/PS	Right-side Glenn + ASD enlargement	Rastelli + ASD closure + Glenn take-down
7	DORV subaortic VSD/IAA/ccTGA	IAA repair + Banding	Double Switch (Senning + ASO)
8	D-TGA/VSD/PS	Right-side Glenn	Rastelli + VSD closure + Glenn take-down
9	DORV remote VSD/PDA/PS	Right-side Glenn + ASD enlargement + PDA ligation	Intracardiac tunnel + RVOT reconstruction + ASD closure + Glenn take-down
10	PA/VSD/ASD	Right-side Glenn	PA/VSD correction + RVOT reconstruction + ASD closure
11	D-TGA/VSD/PS	Right-side BT shunt + atrial enlargement	Rastelli + LVOTO removal
12	DORV remote VSD/SA/TR	Bidirectional Glenn/Atrial septectomy	Intracardiac tunnel + TV valvoplasty + Common atrium repair
13	DORV remote VSD/PAVSD/Atrioventricular discordance/ASD/PDA	Right-side BT shunt + PDA ligation	PA/VSD correction + PA patch enlargement + BT take-down
14	D-TGA/VSD/PS	Right-side Glenn	Rastelli + Glenn take down
15	DORV doubly committed VSD/PS	Left-side BT shunt	Intracardiac tunnel + RVOT reconstruction + VSD enlargement + Glenn take-down

*D-TGA, complete transposition of great arteries; VSD, ventricular septal defect; PS, pulmonary stenosis; PDA, patent ductus arteriosus; DORV, double outlet right ventricle; AVSD, complete atrioventricular septal defect; PA, pulmonary atresia; PS, pulmonary stenosis; ASD, atrial septal defect; IAA, interrupted aortic arch; ccTGA, corrected transposition of great arteries; SA, single atrium; TV, tricuspid valves; TR, tricuspid regurgitation; RVOT, right ventricular outflow tract; LVOT, left ventricular outflow tract*.

**Table 4:** Operative data of each case receiving biventricular conversion in the imbalanced ventricular group.

**Case Number**	**Diagnosis**	**Primary operation**	**Biventricular conversion**
16	Heterotaxy/TAPVC/SV/SA/LSVC	TAPVC repair + bidirectional Glenn	Single ventricular septation + unclosed ASD + Glenn take-down
17	unbalanced AVSD/multiple VSD	Banding + ASD fenestration	AVSD correction + VSD repair + Banding take-down
18	Heterotaxy/AVSD/ASD/PAPVC/PH/MS	PAPVC repair + ASD restriction + Banding	Single ventricular septation + AVSD correction + ASD closure + MV valvoplasty
19	Heterotaxy/TGA/SV/PH	Banding	ASO + SV septation + Banding take-down
20	Unbalanced AVSD/DORV remote VSD/PDA/PH/Tri 21	Banding + PDA ligation	AVSD/DORV repair + pulmonary arterioplasty + Banding take-down
21	Heterotaxy/SV/multiple VSD/ASD/PAPVC/PDA/PH/hypoplasia LV	Banding	SV septation + PAPVC correction + VSD repair/ASD repair/PDA ligation
22	SV (LV dominant)/TGA/PDA/PH	Banding	Switch (ASO) + PDA ligation
23	unbalanced AVSD/PH/PDA	Banding + PDA ligation	AVSD correction + Banding take-down

*TAPVC, total anomalous pulmonary venous connection; PAPVC, partial anomalous pulmonary venous connection; LSVC, left superior vena cava; VSD, ventricular septal defect; ASD, atrial septal defect; MS, mitral stenosis; SV, single ventricle; SA, single atrium; PH, pulmonary hypertension; RVOT, right ventricular outflow tract; ASO, arterial switch operation; HLHS, hypoplastic left heart syndrome; Tri 2, trisomy 21 syndrome*.

The mean time interval between the first operation and biventricular conversion was 20.4 ± 15.8 months in the imbalanced ventricular group. Before conversion, all patients had suitable ventricular size and function for biventricular circulation. Measured by MRI, the mean LVEDV was 33.6 ± 2.1 ml/m^2^ and the mean RVEDV was 98.7 ± 43 ml/m^2^, the systolic volume was 17 ± 5.8 ml/m^2^, and the stroke volume was 29 ± 3.3 ml/m^2^. Measured by catheterization, the mean LVEDP was 9.1 ± 1.9 mmHg. The majority of the patients were classified into mild hypoplasia and received primary conversion, while only two patients had moderate hypoplasia with LVEDV 15–30 ml/m^2^, who received LV recruitment. Early complications included low cardiac output in 2 cases, arrhythmia in 1 case, and renal failure in 1 case. There was no significant difference in bypass time, aortic cross-clamp time, endotracheal intubation time, ICU stays, and in-hospital stays between balanced and imbalanced ventricular groups as can be seen in Table 4 (*P* > 0.05).

### Postoperative Follow-Up

No mortality was observed in 30.2 (median) months follow-up periods (4.2–49.8 months as a range). All patients in both the groups exhibited satisfying life qualities and favorable cardiac function (NYHA I-II grade) during follow-up.

In the balanced ventricular group, one patient had obstruction at the anastomotic site between the right atrial appendage and the superior vena cava after 7 months postoperatively, who required a balloon dilation as reintervention. The patient's echocardiography at the latest follow-up showed the blood flow velocity of superior vena cava was 1.2 m/s, indicating a favorable improvement and maintenance. There was no major complication or adverse events that required reintervention in other patients. The reintervention rate was 6.7%. Moreover, the mean LVEF% at the latest follow-up was 61.3 ± 5.2%, presenting satisfactory cardiac function.

In the imbalanced ventricular group, the only patient requiring reintervention is the one who developed a third-degree atrioventricular block and implanted a pacemaker after 2 months postoperatively. The reintervention rate was 12.5%. Additionally, the mean LVEF% at the latest follow-up was 59.4 ± 5.6%, showing no significant difference with that of the balanced ventricular group (*p* = 0.558, Table 5).

**Table 5:** Postoperative outcomes stratified by different groups.

	**Balanced Ventricular group (*n =* 15)**	**Imbalanced ventricular group (*n =* 8)**	***P*-value**
By-pass time (min)	221.4 ± 87.8	172.8 ± 78.8	0.325
Aortic cross-clamp time (min)	130.3 ± 58.3	111.8 ± 47.0	0.556
Mechanical ventilation time (h)	114.1 ± 71.9	150.0 ± 94.8	0.438
ICU stat length (days)	8.3 ± 3.8	14.8 ± 11.0	0.264
In-hospital stay length (days)	28.4 ± 9.2	29.6 ± 12.8	0.847
Mortality	1 (6.7%)	0	0.455
Reintervention rate	1 (6.7%)	1 (12.5%)	0.636
LVEF (%)	61.3 ± 5.2	59.4 ± 5.6	0.558
NYHA grade (I/II)	12/3	6/2	0.782

*ICU, intensive care unit; LVEF, left ventricle ejection fraction; NYHA, new york heart association function*.

## Discussion

This study reported our single-center experience in biventricular conversion. Pre-operationally, with elaborative evaluations by traditional imaging (echocardiography, enhanced cardiac CT, and cardiac MRI), catheterization, and 3D printing if permitted, 23 patients with complex CHDs were selected to receive a biventricular conversion from single ventricular palliation. Only one mortality occurred because of heart failure. All other cases exhibited an acceptable reintervention rate, excellent cardiac function, and promising life quality at the latest follow-up. Moreover, no significant difference was observed in both early outcomes and follow-up data between balanced and imbalanced ventricular groups, indicating a wide application and promising future of biventricular conversion after careful consideration in specific cases.

The surgical decision-making of complex CHDs depends largely on their anatomical variabilities. Thus far, whether to choose single or biventricular repair is still controversial for patients with severely complex malformations, such as imbalanced ventricles, extreme intracardiac malformation, atrioventricular valve straddling, and conduit reconstructing difficulty. Besides, the surgical strategies in those scenarios are also restrained by age and surgical techniques (15). Most cardiac surgeons may support that a favorable single ventricle is better than two maldeveloped ventricles, as termed as poor biventricular circulation (16–19). Therefore, under most circumstances above, the single ventricular repair is an alteration with relatively higher priority. However, despite the satisfying short- to medium-term results of Fontan operation, severe complications in long term are not rare, such as arrhythmia, thrombosis, protein-losing enteropathy, pulmonary arteriovenous fistula, and heart failure, which might potentially hamper the life qualities of Fontan patients. Reinterventions for those individuals are usually inevitable and costly. Some of the patients with severe adverse events might even have to face heart transplantation as the last but only choice (20–22). In addition, the risk is much higher to perform the Fontan operation when the lesions are combined with pulmonary vein stenosis, right ventricular insufficiency, atrioventricular regurgitation, single pulmonary circulation, or increased pulmonary vascular resistance. In these cases, theoretically, the biventricular correction has the advantage to maintain normal physiology and thus might provide better exercise tolerance, fewer arrhythmias, and better long-term outcomes. Over the years, with unceasing progress in both surgical concepts and techniques, some patients with complex CHDs, who were originally thought to be treated with single ventricular repair, had received biventricular conversion in a few advanced centers and had achieved satisfactory clinical effect (9–12, 23). Corresponding results were shown in our practice.

However, it is also non-negligible that biventricular conversion carries the risk of high mortality and morbidity in short term. Multiple reports have also demonstrated that a considerable number of patients developed significant pulmonary venous hypertension resulting in exercise intolerance and closing the possibility of heart transplantation due to elevation of pulmonary vascular resistance (24–26). From our perspectives, the feasibility and outcomes of biventricular conversion depend largely on the detailed assessment of the intracardiac structure. Thus, we emphasized the role of pre-operational evaluation and patient selection in our study. Over the years, the rapid development of imaging technologies helps to determine the possibility of biventricular conversion. Especially, the new emerging 3D printing technique has shuttled cardiac surgery to a new era with more accurate diagnosis and more visualized simulation. The profit of the 3D printing technique on cardiac surgery has been demonstrated by various research teams (13, 14). Valverde et al. reported an international multicenter study concerning the application of 3D printing on biventricular conversion, emphasizing its outstanding role in refining surgical approaches in complex congenital heart disease (27). Similarly, based on enhanced CT or MRI images, we employed 3D printing technology to accurately print the visualized structure of the heart model before an operation, which helps to further confirm the diagnosis and provide pre-operative simulation with intuitive information. In most of the cases, 3D printing models helped to change the univentricular plans to biventricular and gave much confidence to the surgeons on both surgical decisions and surgical performance. More patients were selected for biventricular correction after we employed the 3D printing technology, which might have been omitted in our previous practice. Additionally, all 10 patients with 3D printing in our study underwent propitious operation with relatively shorter operation time and achieved favorable outcomes during follow-up.

For patients with two well-developed ventricles, with a comprehensive evaluation of anatomic structures pre-operatively, single ventricular palliation could be avoided in most cases in experienced centers. In our study, the balanced ventricular group mainly included two subsets of patients. Due to the restrain of image methods and lack of precise understanding of such techniques in early years, one subgroup was the patients (with an obscure, borderline threshold for biventricular conversion) who were considered to be only suitable for univentricular pathway in our early practice or other centers with less experience, and received Glenn as palliation (66.7%, 10/15). With the advancement in imageology, surgical concepts, and techniques over the years, this subgroup of patients were re-evaluated and re-inspected and received biventricular conversion after taking down the Glenn. Another subgroup was patients with primary biventricular repair in our later and recent practice, who directly avoided the Fontan pathway at the first surgical decision (33.3%, 5/15). In our future practice, with advanced understanding and concepts and after comprehensive evaluations, we would perform the directly biventricular repair (either primary or staged) in selected patients, and there will be no biventricular conversion after Glenn. For patients with functional single ventricle or imbalanced ventricular development, most cardiac surgeons prefer single ventricular palliation due to unpredictable surgical risks of biventricular correction. For this group of patients, the development of each ventricle must be fully evaluated before biventricular conversion. Boston Children's Hospital provided tentative criteria for biventricular conversion in patients with imbalanced ventricles (26), which we also employed in our study. Nathan et al. adopted a staged surgical strategy to promote blood flow through the hypoplastic atrioventricular valves and ventricle *via* ASD restriction (without closing the VSD), thereby significantly improving the success rate of biventricular conversion for the unbalanced common atrioventricular canal (UCAVC) (10). In our study, the same LV recruitment procedure was carried out in 2 selected UCAVC patients (No. 17 and No. 18) with moderate (LV/RV volumes 15–30 ml/m^2^) hypoplasia ventricle, both of whom exhibited excellent cardiac performance after the final biventricular conversion and during follow-up. Cardiovascular angiography and cardiac MRI examination were completed in all cases in the imbalanced ventricular group, providing hemodynamic data as well as ventricular size and function in detail. The mean end-diastolic volume of the hypoplastic ventricle in this group was 33.6 ± 2.1 ml/m^2^, all of which could withstand double ventricular circulation. All the operations went well with no cases of death. However, it was noteworthy that during single ventricular septation, the risk of the third-degree atrioventricular block was still high. There was 1 case of third-degree atrioventricular block after surgery in this group that required permanent pacing implantation.

During the process of biventricular correction from single ventricular palliation, it is often necessary to take down primary surgery, including Glenn anastomosis, B-T shunt, or Banding. When the Glenn is removed, it is required to establish the continuity between the superior vena cava and the right atrium, during which, we took great care to avoid anastomotic stenosis and damage to the sinus node. In elder patients, the continuity of the superior vena cava and the right atrium can be achieved by direct anastomosis or an artificial conduit (28, 29). However, artificial conduit carried the risk of thrombosis, and thus required anticoagulation. Moreover, artificial conduits had no growth potential, for this consideration, it was considered more suitable for elder patients. The mean age of the patients in our study was 4.2 years old. To avoid the possibility of long-term conduit replacement, we tended to use autologous tissues. Autologous tissue from the right atrial appendage was constructed as the posterior wall, and the anterior wall was augmented by the bovine pericardium patch. Only one case of anastomotic stenosis occurred and required further intervention. Our result was consistent with the Glenn take-down experience reported by Baird et al. (30).

Our study was a single-center retrospective study, exploring the feasibility of biventricular conversion from single ventricular palliative in patients with complex congenital heart disease. Based on careful evaluation and meticulous surgical planning, the short-term results were satisfactory in our series and the benefits of biventricular correction were obvious, especially for those with high-risk factors to receive Fontan operation. However, for patients without risk factors, it is still controversial and there is a lack of sufficient evidence to support the efficacy of biventricular correction over Fontan palliation. In our center, an utmost endeavor was made on thorough pre-operational evaluations, and the biventricular correction was applied on those selected patients whenever feasible. Hereafter, we expect to expand our number of cases and to compare the long-term clinical effects through long-term prospective studies.

## Conclusions

Regardless of the development of two ventricles, balanced or imbalanced, the comprehensive evaluation before surgery, especially the 3D printing technique helps to find suitable cases for biventricular conversion. With a tailored surgical strategy according to individual anatomical characteristics, the corresponding biventricular conversion could be carried out more safely in highly selected patients, achieving promising short-term results. However, the long-term outcomes still require further follow-up to be verified.

## Limitations

The biventricular conversion was performed in our center for the recent few years, and thus, the number of patients and the follow-up period were limited. Larger sample size and a longer follow-up span are needed to further illustrate its benefits. MRI, catheterization, and exercise tolerance study should be included in the future follow-up data to fully evaluate the profit and drawbacks of biventricular conversion in the medium to long term. 3D Printing models failed to provide valve information and dynamic changes during a cardiac cycle, which was critically important for patient selection. Different types of congenital heart disease were all included in our study population, and thus, the statistical analysis was not strictly powered since there existed selection bias and unbalanced distribution in our two groups.

## Data Availability Statement

The original contributions presented in the study are included in the article/Supplementary Material, further inquiries can be directed to the corresponding author/s.

## Ethics Statement

The studies involving human participants were reviewed and approved by Shanghai Children Medical Center Ethics Committee. Written informed consent to participate in this study was provided by the participants' legal guardian/next of kin. Written informed consent was obtained from the individual(s), and minor(s)' legal guardian/next of kin, for the publication of any potentially identifiable images or data included in this article.

## Author Contributions

BS and YP: conceptualization and writing–original draft. WL: data curation and statistical analysis. KL: formal analysis. QS: resources. JL: 3D printing technology. ZZ, HW, XH, and JZ: surgical decision and operation. XH and JZ: conception and patients’ follow-up. All authors contributed to the article and approved the submitted version.

## Funding

The study was supported by the Clinical Research Plan of SHDC (SHDC2020CR4093), the General Project of Clinical Research in Health Industry of Shanghai Health Committee (202040337), the Shanghai Municipal Science and Technology Commission (19411963700), and the National Key R&D Program of China (2017YFC1308100).

## Conflict of Interest

The authors declare that the research was conducted in the absence of any commercial or financial relationships that could be construed as a potential conflict of interest.

## Publisher's Note

All claims expressed in this article are solely those of the authors and do not necessarily represent those of their affiliated organizations, or those of the publisher, the editors and the reviewers. Any product that may be evaluated in this article, or claim that may be made by its manufacturer, is not guaranteed or endorsed by the publisher.
